# Tea consumption and major adverse cardiovascular events in coronary heart disease: a non-linear dose–response analysis with joint effect modification by lipoprotein(a) and systemic inflammation — a UK Biobank study

**DOI:** 10.3389/fnut.2026.1847422

**Published:** 2026-05-28

**Authors:** Chunxia Zhang

**Affiliations:** Department of Cardiology, Xingtai People's Hospital, Xingtai, Hebei, China

**Keywords:** coronary heart disease, C-reactive protein, dose–response, effect modification, lipoprotein(a), major adverse cardiovascular events, tea consumption, UK Biobank

## Abstract

**Background:**

Tea consumption is inversely associated with cardiovascular risk in general populations, but evidence from patients with established coronary heart disease (CHD) is limited. Whether lipoprotein(a) [Lp(a)] and C-reactive protein (CRP) modify this association has not been examined.

**Methods:**

From the UK Biobank, 25,306 participants with established CHD were identified (3,773 MACE events over a mean follow-up of 13.9 years). Cox regression with progressive adjustment was performed on 22,012 participants with complete covariate data. Restricted cubic splines (RCS), causal mediation analysis for Lp(a), and three-way interaction tests (tea × Lp(a) × CRP) were performed. A four-quadrant stratification by Lp(a) (cut-off 50 nmol/L) and CRP (cut-off 3 mg/L) was constructed.

**Results:**

RCS analysis identified a non-linear dose–response relationship (*p* < 0.0001), with the lowest MACE risk at approximately 3 cups/day (HR 0.828, 95% CI 0.790–0.867, relative to 0 cups/day). Fully adjusted heavy tea consumption (≥4 cups/day) was associated with reduced risk (HR 0.895, *p* = 0.037). Lp(a) did not mediate the association (ACME *p* > 0.05). A three-way interaction was suggested at the boundary of conventional significance (*p* = 0.050): the low-Lp(a)/high-CRP subgroup showed the strongest benefit (HR 0.670, *p* < 0.001), while the high-Lp(a)/high-CRP subgroup showed none (HR 0.992, *p* = 0.960).

**Conclusion:**

In CHD patients, tea consumption of approximately 3 cups/day is associated with the lowest MACE risk. This association is jointly modified by the Lp(a)–CRP profile, with the greatest benefit in patients whose residual risk is predominantly inflammation-driven.

## 1 Introduction

The global prevalence of cardiovascular diseases doubled between 1990 and 2019, with proportional increases in cardiovascular mortality (1), and this burden continued to escalate through 2022 (2), with atherosclerotic diseases projected to remain the leading cause of cardiovascular death through at least 2050 (3). For patients with established coronary heart disease (CHD), a substantial residual risk of recurrent major adverse cardiovascular events (MACE) persists despite guideline-directed pharmacotherapy (4, 5), and even targeted lipid-lowering strategies beyond statins have yielded inconsistent cardiovascular benefits, as illustrated by the contrasting outcomes of the REDUCE-IT (6) and PROMINENT (7) trials. These gaps underscore the need for complementary, modifiable approaches, among which dietary interventions hold particular promise.

Tea is one of the most widely consumed beverages worldwide. Its bioactive constituents—catechins (notably epigallocatechin gallate), theaflavins, and other polyphenols—attenuate lipid peroxidation, suppress pro-inflammatory cytokine release, and improve endothelial function (8, 9). At the population level, habitual tea consumption has been associated with reduced atherosclerotic cardiovascular disease incidence and all-cause mortality (10). A meta-analysis of 38 prospective cohort data sets reported a pooled relative risk of 0.86 (95% CI 0.79–0.94) for cardiovascular mortality comparing the highest with the lowest consumption categories, with a non-linear dose–response pattern (11). Both black tea (RR 0.85; 95% CI 0.76–0.96) and green tea show dose-dependent protective associations (12), and roughly two cups per day has been proposed as a benefit threshold (13). Whether these general-population findings translate to secondary prevention settings, however, remains uncertain. Patients with established CHD carry a fundamentally different risk profile—higher baseline event rates, widespread statin use, and overt atherosclerotic disease—under which the risk–benefit balance of any dietary exposure may differ substantially, yet evidence in this population remains scarce (14). Moreover, most prior analyses modelled tea intake as a linear variable or used broad categorical comparisons, leaving the non-linear dose–response curve and the optimal protective dose for secondary prevention yet to be defined. Beyond study design, the biological pathways through which tea may confer cardiovascular protection in this high-risk population also remain poorly understood.

Two biomarkers warrant attention as potential effect modifiers. Lipoprotein(a) [Lp(a)] is a genetically determined, causal risk factor for atherosclerotic cardiovascular disease that is largely unresponsive to conventional lipid-lowering therapy (15, 16). As the principal plasma carrier of oxidised phospholipids (OxPLs), Lp(a) activates monocytes and promotes arterial wall inflammation (17, 18). C-reactive protein (CRP), the prototypical marker of chronic low-grade inflammation, is independently associated with coronary heart disease risk and vascular mortality (19–21). Because the OxPLs carried by Lp(a) amplify the inflammatory milieu that CRP reflects, a potential feed-forward loop linking lipid-driven and inflammation-driven atherogenesis may exist (17, 18). When both pathways are concurrently active, the atherogenic substrate against which tea polyphenols must act is qualitatively different from when either pathway operates alone—providing a biological rationale for examining how these two biomarkers may jointly influence tea’s cardiovascular benefit. Whether Lp(a) and CRP, individually or jointly, modify tea’s protective association with MACE has not been examined.

Taken together, the dose–response shape of the tea–MACE relationship in CHD patients has yet to be characterised with flexible modelling approaches, and the roles of Lp(a)—as a potential mediator—and of the combined Lp(a) × CRP profile—as a potential effect modifier—have not been formally evaluated, leaving it unclear which CHD subgroups stand to benefit most from habitual tea consumption (14–16).

The UK Biobank, with its large sample size, prolonged follow-up of up to 15 years, and comprehensive biomarker panel including both Lp(a) and high-sensitivity CRP, provides a unique platform to address these questions. We hypothesised that the tea–MACE association in CHD patients would follow a non-linear dose–response pattern with a plateau at modest intake rather than a continuously linear gradient, and that its magnitude would vary across the joint Lp(a)–CRP biomarker profile, with the greatest benefit in patients whose residual cardiovascular risk is predominantly inflammation-driven. Accordingly, this study aimed to: (1) evaluate the non-linear dose–response relationship between tea consumption and MACE in CHD patients using restricted cubic splines within a Cox proportional hazards framework; (2) test whether Lp(a) mediates the tea–MACE association through formal causal mediation analysis; (3) explore whether the combined Lp(a) × CRP biomarker profile moderates the magnitude of tea’s protective effect using stratified and interaction analyses.

## 2 Methods

### 2.1 Study design and data source

This prospective cohort study used data from the UK Biobank (UKB), a population-based resource that enrolled approximately 502,000 adults aged 40–69 years between 2006 and 2010 at 22 assessment centres across the United Kingdom. At recruitment, participants completed self-administered electronic questionnaires delivered via touchscreen terminals at the assessment centres covering sociodemographic, lifestyle, and dietary factors; underwent physical measurements; and provided biological samples for biochemical assays. The UKB received ethical approval from the North West Multi-Centre Research Ethics Committee (reference 11/NW/0382), and written informed consent was obtained from all participants. Our analysis focused on the subset of participants with established coronary heart disease (CHD) at baseline, constituting a secondary prevention cohort.

### 2.2 Study population

**Figure 1:**
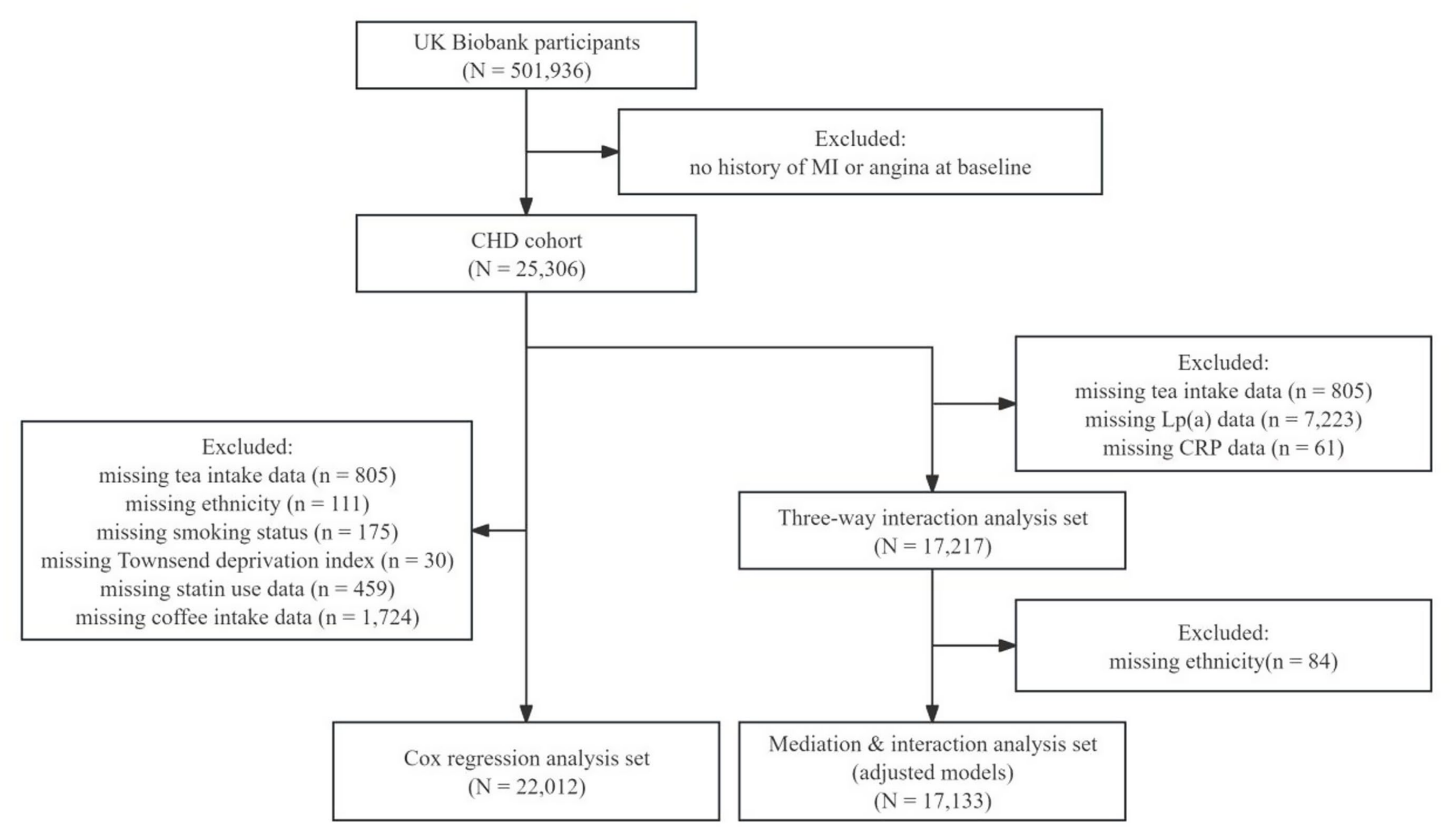
Flow diagram of participant selection. CHD, coronary heart disease; Lp(a), lipoprotein(a); CRP, C-reactive protein; MACE, major adverse cardiovascular events.

Among the 501,936 UKB participants, those with prevalent CHD at baseline were identified on the basis of a documented history of myocardial infarction (MI; UKB Field 131,298, date of first occurrence on or before the baseline assessment) or angina pectoris (Field 131,296, date of first occurrence on or before baseline), yielding 25,306 individuals. From this CHD cohort, two analytic samples were derived depending on the variables required for each analysis (Figure 1).

For Cox proportional hazards regression, participants with missing data on tea intake (Field 1,488 coded as “do not know” or “prefer not to answer”; *n* = 805), ethnicity (*n* = 111), smoking status (*n* = 175), Townsend deprivation index (*n* = 30), statin use (*n* = 459), or coffee intake (*n* = 1,724) were excluded, yielding an analytic sample of 22,012.

For mediation and interaction analyses, a separate sample was derived from the CHD cohort by excluding participants with missing tea intake (*n* = 805), missing lipoprotein(a) [Lp(a)] data (*n* = 7,223), or missing C-reactive protein (CRP) data (*n* = 61), yielding 17,217 individuals for the three-way interaction test and Kaplan–Meier analysis. A further exclusion of those with missing ethnicity (*n* = 84) produced a sample of 17,133 for adjusted mediation and interaction models.

### 2.3 Exposure assessment

Habitual tea consumption was ascertained at baseline using the touchscreen questionnaire item “How many cups of tea do you drink each day?” (Field 1,488). Responses coded as negative values (“do not know” or “prefer not to answer”) were treated as missing. Although this single-item measure has not been formally validated in isolation, short frequency questions of this form are standard in large-scale nutritional epidemiology, and the UK Biobank dietary assessment instruments have demonstrated acceptable reproducibility and concordance with more detailed 24-h dietary recall in prior evaluations (38). Tea intake was modelled in three forms: (1) as a continuous variable (cups/day); (2) as a four-level categorical variable—none (0 cups/day; reference group), light (1 cup/day), moderate (2–3 cups/day), and heavy (≥4 cups/day); and (3) as a binary variable (any tea consumption vs. none). Because Field 1,488 captures aggregate tea intake, the data do not permit differentiation among specific tea types such as black, green, or herbal tea.

### 2.4 Outcome ascertainment

The primary endpoint was a composite of major adverse cardiovascular events (MACE), defined as whichever of the following occurred first after baseline: (1) MI (Field 131,298; ICD-10 I21); (2) stroke (Field 131,368; ICD-10 I64, not specified as haemorrhage or infarction); or (3) cardiovascular death, ascertained from the date-of-death record (Field 40,000) linked to an underlying cause coded within ICD-10 Chapter IX (codes I00–I99, diseases of the circulatory system; Field 40,001). Administrative censoring dates were 31 March 2025 for hospital-based diagnoses (HES data) and 30 November 2024 for mortality records.

### 2.5 Follow-up

Person-time was accrued from the baseline assessment date until the earliest of a MACE event, death from any cause, loss to follow-up (Field 191), or the relevant administrative censoring date, and was expressed in years (dividing by 365.25).

### 2.6 Covariates

Covariate selection was guided by a directed acyclic graph (DAG) constructed to distinguish confounders from variables on the causal pathway. The following confounders were identified and adjusted for in the regression models: age at recruitment (Field 21,003; continuous), sex (Field 31; female as reference), self-reported ethnicity (Field 21,000; grouped as White vs. non-White), Townsend deprivation index (Field 22,189; continuous, with higher values denoting greater area-level deprivation), smoking status (Field 20,116; categorised as never, former, or current), coffee intake (Field 1,498; continuous, cups/day), statin use (Fields 6,153 and 6,177; binary, based on self-reported use of cholesterol-lowering medication), and prevalent diabetes at baseline (Fields 130,706 and 130,708; binary, with either type 1 or type 2 diabetes recorded on or before the baseline date).

LDL cholesterol, blood pressure, and HbA1c were not included in the primary models because these variables may lie on the causal pathway between tea consumption and cardiovascular events, and adjusting for them could introduce over-adjustment bias. BMI and alcohol intake were omitted because their high rates of missing data would have substantially reduced the sample size.

Two biomarkers were evaluated in interaction and mediation analyses. Lp(a) was measured at baseline (Field 30,790; nmol/L), log-transformed as log[Lp(a) + 1] for regression modelling, and dichotomised at the 50 nmol/L threshold recommended by the European Society of Cardiology (15). High-sensitivity CRP (Field 30,710; mg/L) was dichotomised at 3 mg/L, consistent with the American Heart Association threshold for elevated cardiovascular risk (22).

### 2.7 Statistical analysis

Baseline characteristics across the four tea intake categories were compared using one-way ANOVA or the Kruskal–Wallis test for continuous variables and the chi-squared test for categorical variables. Continuous data are reported as mean ± SD or median [IQR]; categorical data as counts (percentages).

To estimate the overall association between tea consumption and MACE and to assess how much of any observed association was explained by confounding, we fitted three sequential Cox proportional hazards models with progressive covariate adjustment. Hazard ratios (HRs) with 95% confidence intervals (CIs) were estimated as follows: Model 1 was unadjusted; Model 2 adjusted for age, sex, and ethnicity; Model 3 further adjusted for smoking status, Townsend deprivation index, statin use, diabetes, and coffee intake. The proportional hazards assumption was evaluated using scaled Schoenfeld residuals. Missing covariate data were handled by complete-case analysis. Baseline characteristics of participants with and without available Lp(a) were compared using standardised mean differences (Supplementary Table S1).

To examine whether the tea–MACE association followed a non-linear dose–response pattern rather than being adequately summarised by a single linear slope, we modelled tea intake flexibly using restricted cubic splines (RCS) with four knots within the Cox framework, adjusting for covariates. Non-linearity was assessed by a Wald test on the higher-order spline terms. HRs derived from the same fitted curve are reported under two reference frames: the sample mean (3.7 cups/day) when describing the overall shape, and 0 cups/day (non-consumption) when reporting the nadir estimate for comparability with the categorical analyses.

To test whether any observed protective effect of tea operated by lowering Lp(a) levels (i.e., whether Lp(a) lies on the causal pathway), we performed causal mediation analysis using the R mediation package with 1,000 bootstrap iterations. The mediator model was a linear regression of log-transformed Lp(a) on tea intake adjusted for age, sex, and ethnicity; the outcome model was a logistic regression of MACE on tea intake and log-transformed Lp(a) with the same covariates. Logistic regression was used in place of a Cox model owing to technical constraints in the mediation package; given the MACE event rate of approximately 15%, the resulting odds ratios may modestly overestimate hazard ratios, but the direction and significance of the average causal mediation effect (ACME) remain interpretable. Three exposure contrasts were evaluated: per 1 cup/day, heavy vs. none, and moderate vs. none.

To test whether the magnitude of tea’s protective association differed according to patients’ Lp(a) and CRP status (i.e., whether either biomarker acts as an effect modifier rather than a mediator), we performed a sequence of interaction analyses. Two-way multiplicative interactions between tea intake (binary) and each biomarker group (Lp(a) high vs. low; CRP high vs. low) were tested in Cox models adjusted for age, sex, and ethnicity. A three-way interaction term (tea × Lp(a) group × CRP group) was evaluated by comparing a saturated model against one containing only two-way interactions using a likelihood ratio test (N = 17,217; unadjusted). Tea intake was dichotomised (any vs. none) for this analysis to ensure adequate event counts within each cell of the eight-cell cross-classification and to maintain the numerical stability of the interaction model. Stratified Cox analyses were then conducted within subgroups defined by Lp(a) level and CRP level separately, as well as within a joint four-quadrant classification cross-tabulating Lp(a) (≥50 vs. <50 nmol/L) with CRP (≥3 vs. <3 mg/L). Cumulative MACE incidence across the four quadrants was estimated using the Kaplan–Meier method.

All analyses were conducted in R (version 4.3.2). Two-sided *p* values below 0.05 were considered statistically significant.

## 3 Results

### 3.1 Study population

Of the 501,936 UKB participants, 25,306 had established CHD at baseline. After excluding those with missing tea intake data (n = 805), 24,501 individuals were retained for baseline characterisation (Table 1). Tea consumption was distributed as follows: none, 3,705 (15.1%); light, 1,822 (7.4%); moderate, 6,958 (28.4%); and heavy, 12,016 (49.0%). Over a mean follow-up of 13.9 ± 4.2 years (median 15.5 years), 3,773 MACE events (15.4%) accrued. After further exclusion of participants with missing covariate data, 22,012 were included in the Cox regression analyses and 17,133 in the adjusted mediation and interaction analyses (Figure 1). Baseline characteristics were well balanced between participants with and without available Lp(a) (all |SMD| < 0.10; Supplementary Table S1).

**Table 1:** Baseline characteristics of CHD patients according to tea intake category.

Variables	None	Light	Moderate	Heavy	*p*
*N*	3,705	1,822	6,958	12,016	
Age (years), mean (SD)	61.35 (6.15)	61.14 (6.51)	62.12 (5.94)	62.11 (5.86)	<0.001
Male sex, *n* (%)	2,474 (66.8)	1,328 (72.9)	4,831 (69.4)	8,273 (68.8)	<0.001
Non-white ethnicity, *n* (%)	104 (2.8)	189 (10.4)	712 (10.3)	426 (3.6)	<0.001
Townsend index, median [IQR]	−1.15 [−3.21, 2.20]	−1.57 [−3.36, 1.78]	−1.70 [−3.43, 1.42]	−1.49 [−3.28, 1.87]	<0.001
BMI (kg/m^2^), mean (SD)	30.31 (5.46)	29.40 (5.00)	29.17 (4.99)	29.38 (4.96)	<0.001
Waist circumference (cm)	100.20 (13.97)	98.79 (13.29)	97.71 (13.05)	98.10 (13.35)	<0.001
SBP (mmHg), mean (SD)	140.30 (19.57)	141.07 (19.81)	140.82 (19.77)	140.42 (19.75)	0.341
DBP (mmHg), mean (SD)	79.17 (10.88)	79.86 (10.56)	79.26 (10.76)	78.96 (10.86)	0.010
TC (mmol/L), mean (SD)	4.71 (1.12)	4.64 (1.06)	4.64 (1.09)	4.62 (1.08)	<0.001
LDL-C (mmol/L), mean (SD)	2.86 (0.83)	2.81 (0.78)	2.80 (0.79)	2.79 (0.79)	<0.001
TG (mmol/L), median [IQR]	1.77 [1.25, 2.48]	1.70 [1.20, 2.42]	1.67 [1.17, 2.40]	1.64 [1.17, 2.34]	<0.001
Lp(a) (nmol/L), median [IQR]	22.20 [9.26, 68.90]	27.92 [10.90, 78.58]	25.44 [10.57, 77.47]	24.20 [10.04, 71.94]	0.003
CRP (mg/L), median [IQR]	1.68 [0.84, 3.69]	1.48 [0.75, 3.08]	1.48 [0.73, 3.11]	1.64 [0.80, 3.38]	<0.001
HbA1c (%), mean (SD)	5.90 (0.98)	5.81 (0.84)	5.83 (0.90)	5.83 (0.89)	0.001
Creatinine (μmol/L), median [IQR]	75.90 [65.90, 87.00]	77.80 [68.10, 89.20]	77.40 [67.20, 89.10]	78.60 [67.90, 89.90]	<0.001
Platelet (×10^9^/L), median [IQR]	236.40 [201.00, 278.00]	229.15 [196.40, 267.90]	229.30 [195.20, 268.00]	234.80 [199.00, 275.90]	<0.001
Coffee (cups/day), mean (SD)	4.01 (3.21)	2.72 (2.12)	1.97 (1.78)	1.41 (1.88)	<0.001
Smoking status, *n* (%)					<0.001
Never	1,219 (33.1)	723 (39.9)	2,903 (42.0)	4,391 (36.8)	
Former	1814 (49.2)	891 (49.2)	3,370 (48.8)	5,911 (49.6)	
Current	651 (17.7)	196 (10.8)	633 (9.2)	1,619 (13.6)	
Statin use, *n* (%)	2,945 (80.2)	1,483 (82.2)	5,630 (81.6)	9,610 (80.7)	0.154
Diabetes, *n* (%)	600 (16.2)	244 (13.4)	971 (14.0)	1,592 (13.2)	<0.001
Follow-up (years), mean (SD)	13.57 (4.39)	13.96 (4.22)	14.00 (4.01)	13.84 (4.19)	<0.001
MACE events, n (%)	619 (16.7)	266 (14.6)	1,027 (14.8)	1861 (15.5)	0.046

BMI, body mass index; SBP, systolic blood pressure; DBP, diastolic blood pressure; TC, total cholesterol; LDL-C, low-density lipoprotein cholesterol; TG, triglycerides; Lp(a), lipoprotein(a); CRP, C-reactive protein; HbA1c, glycated haemoglobin. Tea categories: None, 0 cups/day (reference); Light, 1 cup/day; Moderate, 2–3 cups/day; Heavy, ≥4 cups/day.

### 3.2 Baseline characteristics

Table 1 presents the baseline characteristics according to tea intake category. Non-tea-drinkers had the highest mean BMI (30.31 ± 5.46 kg/m^2^), the highest median CRP (1.68 [0.84, 3.69] mg/L), the highest HbA1c (5.90 ± 0.98%), and the greatest proportion of current smokers (17.7%) and diabetic individuals (16.2%). Coffee consumption was inversely related to tea intake, declining from 4.01 ± 3.21 cups/day among non-drinkers to 1.41 ± 1.88 cups/day among heavy tea drinkers (*p* < 0.001). Median Lp(a) was lowest in non-drinkers (22.20 [9.26, 68.90] nmol/L) and highest in light drinkers (27.92 [10.90, 78.58] nmol/L; *p* = 0.003). Systolic blood pressure and statin use did not differ significantly across groups (*p* = 0.341 and *p* = 0.154, respectively). Although LDL cholesterol showed a statistically significant trend (*p* < 0.001), the absolute differences across groups were small (range 2.79–2.86 mmol/L).

### 3.3 Tea consumption and MACE risk

In the unadjusted model (Table 2), all three tea-drinking categories had lower MACE hazards than non-drinkers: light consumption, HR 0.841 (95% CI 0.725–0.975; *p* = 0.022); moderate, 0.862 (0.777–0.955; *p* = 0.005); and heavy, 0.904 (0.823–0.994; *p* = 0.037). These associations strengthened after adjustment for age, sex, and ethnicity (Model 2), with moderate consumption yielding the largest point estimate of benefit (HR 0.816, 0.736–0.906; *p* < 0.001).

**Table 2:** Association between tea intake and MACE risk: Cox proportional hazards models.

Variables	Model 1		Model 2		Model 3	
	HR (95% CI)	*p*	HR (95% CI)	*p*	HR (95% CI)	*p*
Continuous	1.002 (0.992–1.013)	0.682	1.002 (0.992–1.013)	0.662	0.995 (0.984–1.007)	0.430
Light	0.841 (0.725–0.975)	**0.022**	0.818 (0.705–0.949)	**0.008**	0.895 (0.770–1.04)	0.149
Moderate	0.862 (0.777–0.955)	**0.005**	0.816 (0.736–0.906)	**< 0.001**	0.898 (0.805–1.002)	0.054
Heavy	0.904 (0.823–0.994)	**0.037**	0.866 (0.788–0.952)	**0.003**	0.895 (0.806–0.993)	**0.037**

HR, hazard ratio; CI, confidence interval. Tea categories: None (0 cups/day, reference); Light (1 cup/day); Moderate (2–3 cups/day); Heavy (≥4 cups/day). Continuous, HR per 1 cup/day increment. Model 1: unadjusted. Model 2: age, sex, ethnicity. Model 3: Model 2 + smoking status, Townsend deprivation index, statin use, diabetes at baseline, and coffee intake. Bold values indicate statistically significant associations (*p* < 0.05).

In the fully adjusted model (Model 3), heavy tea consumption remained associated with a statistically significant 10.5% reduction in MACE risk (HR 0.895, 0.806–0.993; *p* = 0.037). The estimate for moderate consumption was of similar magnitude but fell just short of conventional significance (HR 0.898, 0.805–1.002; *p* = 0.054). When tea was entered as a continuous variable, the per-cup HR was close to unity in all three models (Model 3: HR 0.995, 0.984–1.007; *p* = 0.430), consistent with a non-linear rather than strictly linear dose–response pattern. The Schoenfeld residuals test confirmed that the proportional hazards assumption was met.

### 3.4 Dose–response relationship

**Figure 2:**
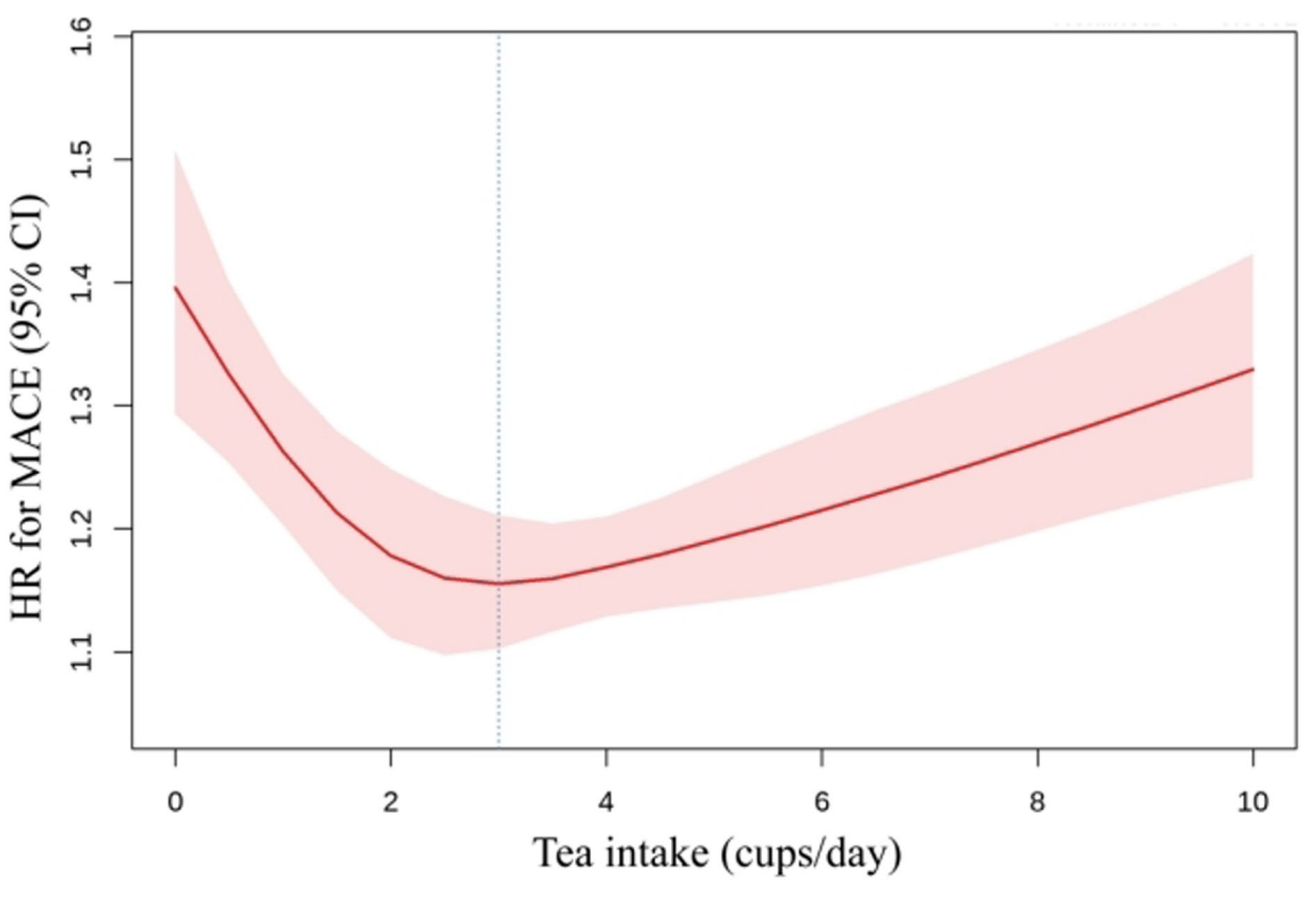
Non-linear dose–response relationship between tea intake and MACE risk in CHD patients. Restricted cubic splines (4 knots) adjusted for age, sex, and ethnicity. The sample mean intake (3.7 cups/day) serves as the internal reference of the spline; the HR relative to 0 cups/day is obtained by re-centring the same fitted curve. The lowest risk was observed at approximately 3 cups/day, corresponding to HR = 0.828 (95% CI: 0.790–0.867) relative to 0 cups/day. Nonlinear *p* < 0.0001.

The RCS analysis demonstrated a non-linear inverse relationship between tea intake and MACE risk (non-linearity *p* < 0.0001; Figure 2). The curve reached its lowest point at approximately 3 cups/day and plateaued thereafter. Relative to 0 cups/day, this nadir corresponded to a 17% lower MACE risk (HR 0.828, 95% CI 0.790–0.867).

### 3.5 Mediation analysis

Table 3 summarises the mediation analysis results. In all three exposure contrasts, the ACME through Lp(a) was negligible and non-significant. For the continuous contrast (per 1 cup/day), the ACME was 5.18 × 10^−6^ (95% CI −2.30 × 10^−5^ to 3.68 × 10^−5^; *p* = 0.728). For heavy versus none, the ACME was 8.88 × 10^−5^ (*p* = 0.598) despite a significant ADE (*p* = 0.002). For moderate versus none, the ACME was 4.21 × 10^−4^ (*p* = 0.148), while the total effect was significant (*p* < 0.001). These findings indicate that Lp(a) does not mediate the association between tea consumption and MACE. Whether Lp(a) instead acts as an effect modifier was examined in subsequent interaction analyses.

**Table 3:** Mediation analysis of Lp(a) in the association between tea intake and MACE.

Comparison	ACME (95% CI)	ACME P	ADE P	Total effect P
Continuous (per 1 cup/day)	5.18 × 10^−6^ (−2.30 × 10^−5^, 3.68 × 10^−5^)	0.728	0.926	0.926
Heavy vs. None	8.88 × 10^−5^ (−2.45 × 10^−4^, 4.93 × 10^−4^)	0.598	0.002	0.002
Moderate vs. None	4.21 × 10^−4^ (−1.18 × 10^−4^, 1.27 × 10^−3^)	0.148	<0.001	<0.001

ACME, average causal mediation effect; ADE, average direct effect; CI, confidence interval; Lp(a), lipoprotein(a). Restricted sample (N = 17,133), adjusted for age, sex, and ethnicity. Bootstrap = 1,000 iterations.

### 3.6 Interaction and joint stratification analyses

A three-way interaction among tea intake (binary), Lp(a) group, and CRP group was suggested at the boundary of conventional significance (LRT *p* = 0.050), indicating that the cardiovascular benefit of tea consumption may vary depending on the combined Lp(a)–CRP profile.

**Figure 3:**
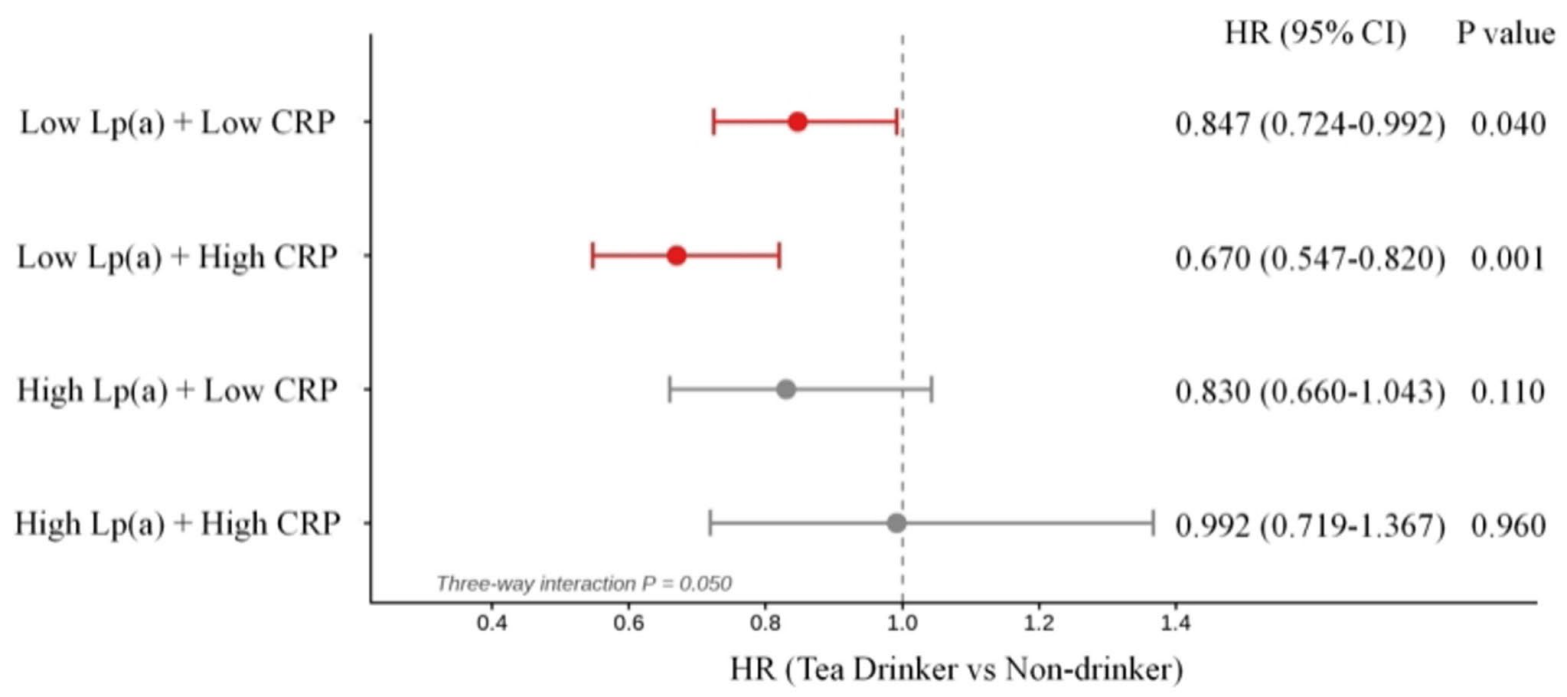
Forest plot of MACE risk associated with tea consumption across joint Lp(a) and CRP subgroups. Hazard ratios compare tea drinkers with non-drinkers within each subgroup, adjusted for age, sex, and ethnicity (*N* = 17,133). Lp(a) cutoff: 50 nmol/L; CRP cutoff: 3 mg/L. Error bars represent 95% confidence intervals.

Table 4 and Figure 3 present the four-quadrant analysis. Among participants with low Lp(a) and high CRP, tea drinking was associated with a 33% lower MACE risk compared with non-drinkers (HR 0.670, 95% CI 0.547–0.820; *p* < 0.001; *N* = 3,237; 565 events)—the strongest reduction observed in any subgroup. The low-Lp(a)/low-CRP subgroup also showed a significant association (HR 0.847, 0.724–0.992; *p* = 0.040; *N* = 8,215; 1,131 events). In the high-Lp(a)/low-CRP group, the point estimate favoured tea drinkers but did not reach significance (HR 0.830, 0.660–1.043; *p* = 0.110). In the high-Lp(a)/high-CRP group, no association was apparent (HR 0.992, 0.719–1.367; *p* = 0.960).

**Table 4:** Tea consumption and MACE risk across joint Lp(a) and CRP subgroups.

Subgroup	*N*	Events	HR (95% CI)	*p*
Low Lp(a) + Low CRP	8,215	1,131	0.847 (0.724–0.992)	0.040
Low Lp(a) + High CRP	3,237	565	0.670 (0.547–0.820)	<0.001
High Lp(a) + Low CRP	4,126	581	0.830 (0.660–1.043)	0.110
High Lp(a) + High CRP	1,555	305	0.992 (0.719–1.367)	0.960

HR, hazard ratio (tea drinker vs. non-drinker); CI, confidence interval; Lp(a), lipoprotein(a); CRP, C-reactive protein. Adjusted for age, sex, and ethnicity. Lp(a) cut-off: 50 nmol/L; CRP cut-off: 3 mg/L. Three-way interaction (tea × Lp(a) × CRP): *p* = 0.050.

### 3.7 Cumulative MACE incidence by Lp(a)–CRP profile

**Figure 4:**
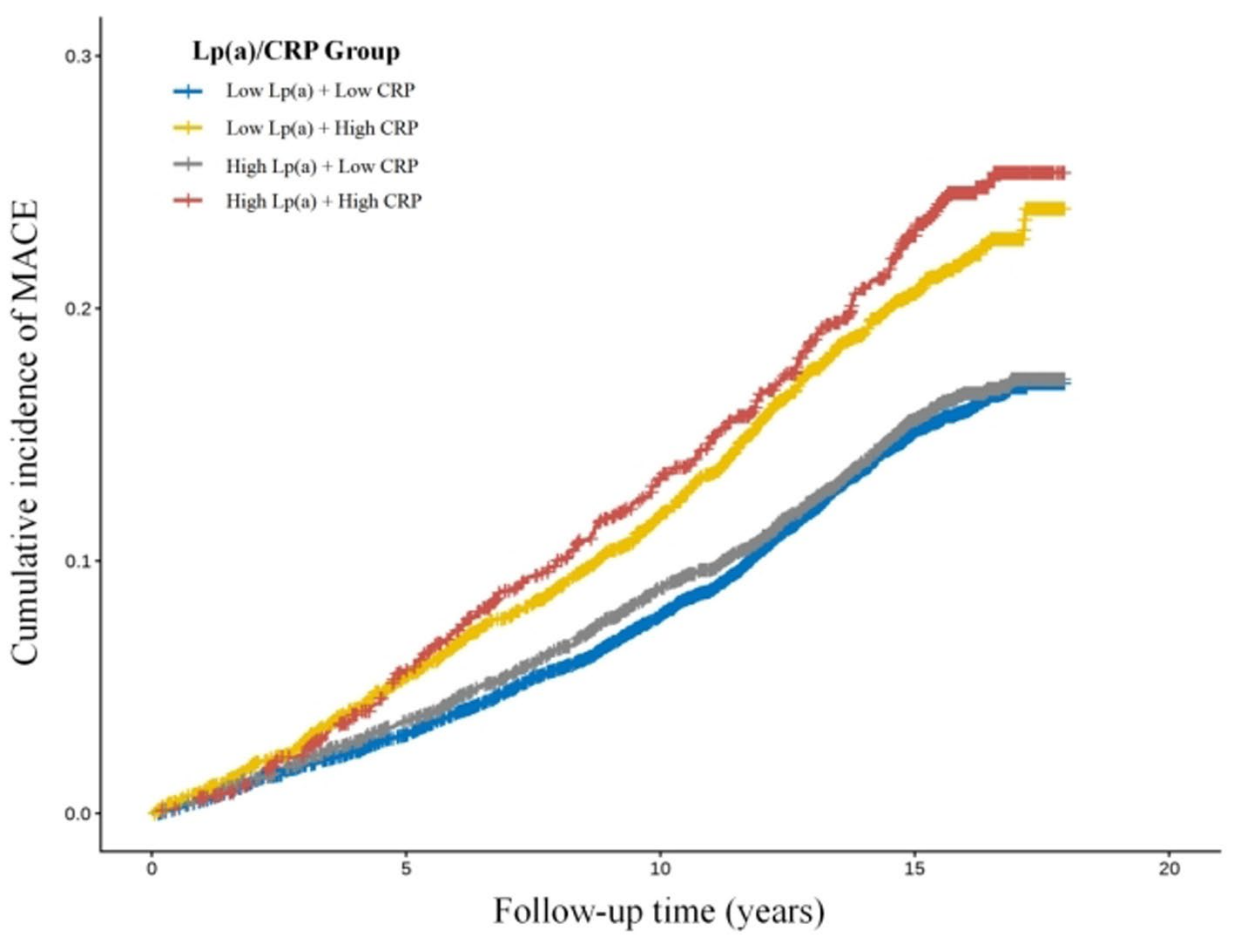
Cumulative incidence of MACE by joint Lp(a) and CRP status in CHD patients. Kaplan–Meier estimates of cumulative MACE incidence stratified by joint Lp(a) (≥50 vs. <50 nmol/L) and CRP (≥3 vs. <3 mg/L) status (*N* = 17,217). Curves represent all-comer risk within each subgroup, irrespective of tea consumption.

Kaplan–Meier curves (Figure 4) confirmed that cardiovascular risk in this CHD cohort was stratified by the combined Lp(a)–CRP profile. Participants in the high-Lp(a)/high-CRP group had the steepest rise in cumulative MACE incidence, reaching approximately 27% at the end of follow-up. The low-Lp(a)/low-CRP group had the lowest incidence (~17%), and the two discordant groups tracked intermediate trajectories. Separation among the curves became apparent around year 5 and widened progressively thereafter. Taken together, habitual tea consumption showed a non-linear inverse association with MACE risk that plateaued beyond approximately 3 cups/day; this association was not mediated by Lp(a) but varied in magnitude across Lp(a)–CRP strata, with the greatest benefit observed in the low-Lp(a)/high-CRP subgroup.

## 4 Discussion

In this prospective cohort of 25,306 patients with established CHD from the UK Biobank, followed for a mean of 13.9 years, we found a significant non-linear inverse association between habitual tea consumption and MACE risk. Restricted cubic spline analysis identified a risk nadir at approximately 3 cups/day (HR 0.828, 95% CI 0.790–0.867, relative to 0 cups/day; non-linearity *p* < 0.0001), and heavy tea consumption (≥4 cups/day) remained independently associated with a 10.5% reduction in MACE risk after full covariate adjustment (HR 0.895, 0.806–0.993; *p* = 0.037). Causal mediation analysis demonstrated that Lp(a) does not mediate the tea–MACE relationship, while a three-way interaction among tea intake, Lp(a), and CRP (LRT *p* = 0.050) indicated that the protective effect of tea varies according to the combined biomarker profile, with the greatest benefit observed in the low-Lp(a)/high-CRP subgroup (HR 0.670; *p* < 0.001).

Previous meta-analyses conducted in general-population cohorts have consistently reported inverse associations between tea consumption and cardiovascular outcomes, with estimated risk reductions of 8–12% per 3-cup/day increment (14, 23). Our RCS-derived nadir HR of 0.828 is directionally concordant with these estimates but provides additional information that linear models cannot capture: specifically, the dose–response curve plateaus beyond 3 cups/day, explaining why tea entered as a continuous per-cup variable yielded a near-null association in all three models. These findings align with Yang et al., who observed that black tea intake exceeding 4–6 cups/day may not confer further benefit (12), and with Keller et al.’s proposed threshold of approximately 2 cups/day (13). The attenuation of effect estimates from the age- and sex-adjusted model to the fully adjusted model is consistent with partial healthy-user confounding, a well-recognised phenomenon in observational dietary research (24). Nonetheless, heavy tea consumption remained significantly associated with lower MACE risk after full covariate adjustment, suggesting that lifestyle confounding does not wholly account for the association.

A Mendelian randomisation study by Chen et al. found no causal association between genetically predicted tea consumption and CVD in the general population (25). This null result, however, reflects average genetic effects across unselected populations and does not preclude subgroup-specific benefits in high-risk secondary prevention cohorts, where the pathophysiological substrate—established atherosclerosis, endothelial dysfunction, and chronic inflammation—amplifies the relevance of tea polyphenols’ anti-inflammatory mechanisms (8, 9).

The ACME was non-significant across all three exposure contrasts (*p* = 0.148–0.728), while the average direct effect for both the heavy-versus-none (*p* = 0.002) and moderate-versus-none (*p* < 0.001) comparisons remained significant. This pattern indicates that tea’s cardioprotective effect appears to operate independently of the Lp(a) pathway. The null mediation is biologically coherent: Lp(a) concentrations are predominantly genetically determined and minimally responsive to dietary interventions. The EAS consensus statement confirms that specific Lp(a)-lowering therapies remain in clinical development and that existing lifestyle modifications have limited impact on Lp(a) levels (26). Consistent with this, Santos et al. found no justification for higher intakes of various dietary supplements to lower Lp(a) (27), and Enkhmaa et al. reported that dietary effects on Lp(a) are modest and heterogeneous (28). These observations collectively redirect attention from mediation to moderation: rather than acting through Lp(a), tea’s benefit varies in magnitude according to the patient’s Lp(a) burden.

The four-quadrant analysis revealed a clear gradient: the strongest protective association was observed in the low-Lp(a)/high-CRP subgroup (HR 0.670, *p* < 0.001), followed by the low-Lp(a)/low-CRP group (HR 0.847, *p* = 0.040), while neither high-Lp(a) subgroup showed a significant association. This pattern has a plausible mechanistic basis.

Patients with low Lp(a) and elevated CRP represent a phenotype in which cardiovascular risk is predominantly inflammation-driven, a milieu in which the anti-inflammatory properties of tea-derived polyphenols can exert their maximal effect. Much of the mechanistic literature on this effect has focused on EGCG and its inhibition of NF-κB signalling (29); however, tea consumption in this UK-based cohort is predominantly in the form of black tea, in which catechins are largely oxidised during fermentation to theaflavins and thearubigins, compounds that likewise exhibit anti-inflammatory and antioxidant activity in experimental models but whose bioavailability and *in vivo* potency relative to EGCG remain incompletely characterised (39, 40). The common British practice of adding milk may further modify polyphenol bioavailability (41). Because the UK Biobank questionnaire does not capture tea type or preparation, we regard the specific molecular pathway as underdetermined in our data, while the broader class of tea-derived anti-inflammatory polyphenols provides a coherent framework for the observed subgroup pattern. In contrast, elevated Lp(a) introduces an OxPL-driven inflammatory burden that is genetically fixed and not modifiable by dietary anti-oxidants (17, 18), effectively attenuating tea’s benefit. In the high-Lp(a)/high-CRP subgroup, the elevated CRP may itself be partly driven by the Lp(a)–OxPL pathway rather than representing an independent inflammatory source; consequently, even in the presence of high CRP, tea polyphenols acting downstream on cytokine signalling cannot counteract the upstream lipoprotein-mediated inflammation.

These findings extend work by Arnold et al., who demonstrated in the BiomarCaRE project that Lp(a) was associated with recurrent CHD events only in the presence of residual inflammatory risk among secondary prevention patients (30), and by Zhang et al., who showed that co-elevation of Lp(a) and CRP synergistically increases ASCVD and all-cause mortality (31). Our study adds a dietary dimension to this Lp(a)–CRP joint risk framework: the same biomarker profile that identifies patients at highest pharmacological residual risk also predicts which patients derive the greatest benefit from tea consumption. Taken together, these results suggest that Lp(a) and CRP modulate the magnitude of tea’s protective effect rather than its presence or absence, supporting a general dietary recommendation of approximately 3 cups/day with the understanding that the expected benefit will differ according to the individual’s biomarker profile. These subgroup findings should be regarded as hypothesis-generating given the borderline interaction *p* value, and warrant prospective replication in independent secondary prevention cohorts before informing clinical decisions.

Given an observed MACE incidence of approximately 15% over 13.9 years in this cohort, the 17% relative risk reduction at the dose–response nadir (relative to non-consumption) translates to an absolute risk reduction on the order of 2–3 percentage points, and the 10.5% reduction observed with fully adjusted heavy consumption to approximately 1.5 percentage points—magnitudes that are clinically meaningful for a widely accessible, low-cost dietary exposure. From a clinical perspective, these results suggest that CHD patients with low Lp(a) and elevated CRP—a profile readily identifiable using existing clinical biomarker panels—represent optimal candidates for tea-based dietary recommendations in secondary prevention. For high-Lp(a) subgroups where tea alone appears insufficient, future trials combining emerging Lp(a)-targeted therapies (e.g., pelacarsen (32)) with dietary anti-inflammatory strategies may be needed to achieve meaningful risk reduction (33).

Strengths of this study include the large sample size and extended follow-up (mean 13.9 years) providing high statistical power, a progressive three-model adjustment strategy that transparently quantifies the contribution of confounding, and RCS modelling that avoids the linearity assumption limiting prior tea–CVD analyses. Bootstrap mediation with 1,000 iterations ensured the robustness of the ACME null finding.

Several limitations warrant consideration. Tea intake was assessed by self-report at a single baseline time-point, introducing recall bias and precluding capture of long-term dietary changes; such measurement error typically attenuates associations, suggesting our HRs may underestimate the true effect (34). This concern is particularly relevant in a secondary prevention cohort, as CHD patients often modify lifestyle behaviours after diagnosis in response to medical advice or heightened health awareness, so baseline tea intake may not reflect habitual consumption over the subsequent 13.9 years of follow-up. The UKB questionnaire does not distinguish among tea types, and catechin content varies substantially across black, green, and herbal varieties (35). The first-occurrence MI field cannot capture recurrent events in patients with prior MI, potentially underestimating MACE incidence. The mediation analysis used logistic regression to approximate Cox regression; although this is a recognised approach when event rates are moderate (~15%), odds ratios may modestly overestimate hazard ratios. The mediation and interaction analyses were restricted to participants with available Lp(a) and CRP data, excluding approximately 29% of the CHD cohort; baseline characteristics were comparable between those with and without available Lp(a) (Supplementary Table S1), arguing against substantial selection bias, although residual effects from unmeasured factors cannot be excluded. Despite progressive adjustment, residual confounding from unmeasured factors—including brewing methods, additions (milk, sugar), and co-occurring dietary patterns—cannot be excluded. Finally, the UK Biobank is subject to healthy volunteer bias (36), although risk-factor associations in this cohort have been shown to be generally generalisable to representative populations (37). Finally, dichotomising tea intake in the three-way interaction analysis may have underestimated effect modification operating in a dose-dependent manner.

## 5 Conclusion

In patients with established CHD, habitual tea consumption of approximately 3 cups/day is associated with the lowest MACE risk. This benefit is not mediated by Lp(a) but is jointly modified by the combined Lp(a)–CRP profile, with the greatest risk reduction observed in patients whose residual risk is predominantly inflammation-driven. Future priorities include Mendelian randomisation analyses in secondary prevention subgroups, tea-type-stratified studies, and prospective validation of the Lp(a) × CRP moderating framework.

## Data Availability

Publicly available datasets were analyzed in this study. The UK Biobank is an open-access resource available to approved researchers. Data access can be requested at https://www.ukbiobank.ac.uk. The authors do not have permission to publicly share the raw data, as redistribution is prohibited under the terms of the UK Biobank Access Agreement.
