# Supplementary material for: Tea consumption and major adverse cardiovascular events in coronary heart disease: a non-linear dose–response analysis with joint effect modification by lipoprotein(a) and systemic inflammation — a UK Biobank study
Source: Front Nutr. 2026 May 28;13:1847422. doi: 10.3389/fnut.2026.1847422 (PMC13255769; doi:10.3389/fnut.2026.1847422)
Supplement: Supplementary file 1 [file Table_1.docx]

**Supplementary Table S1.** Comparison of baseline characteristics between CHD participants with and without available lipoprotein(a) measurements.

|  | Overall | Lp(a) available | Lp(a) missing | p | SMD |
| --- | --- | --- | --- | --- | --- |
| N | 24,501 | 17,278 | 7,223 |  |  |
| Age (years), mean (SD) | 61.93 (5.99) | 61.97 (5.96) | 61.82 (6.05) | 0.064 | 0.026 |
| Townsend index, median [IQR] | -1.51 [-3.32, 1.80] | -1.55 [-3.34, 1.74] | -1.39 [-3.28, 1.97] | 0.018 | 0.03 |
| BMI (kg/m²), mean (SD) | 29.46 (5.06) | 29.41 (5.05) | 29.59 (5.09) | 0.011 | 0.036 |
| Waist circumference (cm) | 98.36 (13.38) | 98.23 (13.34) | 98.68 (13.47) | 0.017 | 0.033 |
| SBP (mmHg), mean (SD) | 140.56 (19.73) | 140.45 (19.79) | 140.85 (19.60) | 0.167 | 0.02 |
| DBP (mmHg), mean (SD) | 79.14 (10.81) | 79.11 (10.80) | 79.23 (10.84) | 0.446 | 0.011 |
| TC (mmol/L), mean (SD) | 4.64 (1.09) | 4.63 (1.09) | 4.67 (1.06) | 0.011 | 0.04 |
| LDL-C (mmol/L), mean (SD) | 2.80 (0.80) | 2.80 (0.80) | 2.82 (0.78) | 0.043 | 0.032 |
| TG (mmol/L), median [IQR] | 1.67 [1.18, 2.38] | 1.68 [1.18, 2.39] | 1.65 [1.17, 2.36] | 0.095 | 0.004 |
| CRP (mg/L), median [IQR] | 1.58 [0.78, 3.32] | 1.59 [0.79, 3.31] | 1.57 [0.76, 3.36] | 0.535 | 0.011 |
| HbA1c (%), mean (SD) | 5.84 (0.91) | 5.83 (0.90) | 5.86 (0.91) | 0.018 | 0.035 |
| Creatinine (μmol/L), median [IQR] | 77.80 [67.40, 89.20] | 77.80 [67.30, 89.30] | 77.80 [67.80, 88.90] | 0.913 | 0.012 |
| Platelet (×10⁹/L), median [IQR] | 233.00 [198.00, 273.00] | 233.00 [198.00, 273.00] | 233.35 [198.90, 273.30] | 0.497 | 0.007 |
| Coffee (cups/day), mean (SD) | 2.09 (2.33) | 2.09 (2.33) | 2.09 (2.33) | 0.987 | <0.001 |
| Follow-up (years), mean (SD) | 13.86 (4.18) | 13.89 (4.13) | 13.77 (4.29) | 0.035 | 0.029 |
| Tea (cups/day) median [IQR] | 3.00 [2.00, 5.00] | 3.00 [2.00, 5.00] | 3.00 [2.00, 5.00] | 0.89 | 0.003 |
| Male sex, n (%) | 16906 (69.0) | 11960 (69.2) | 4946 (68.5) | 0.256 | 0.016 |
| Non-white ethnicity, n (%) | 1431 ( 5.9) | 1082 ( 6.3) | 349 ( 4.8) | <0.001 | 0.063 |
| Smoking status, n (%) |  |  |  | 0.015 | 0.041 |
| Never | 9236 (38.0) | 6610 (38.5) | 2626 (36.7) |  |  |
| Former | 11986 (49.3) | 8359 (48.7) | 3627 (50.6) |  |  |
| Current | 3099 (12.7) | 2187 (12.7) | 912 (12.7) |  |  |
| Statin use, n (%) | 19668 (81.0) | 13822 (80.0) | 5846 (83.4) | <0.001 | 0.089 |
| Diabetes, n (%) | 3407 (13.9) | 2383 (13.8) | 1024 (14.2) | 0.439 | 0.011 |
| MACE events, n (%) | 3773 (15.4) | 2609 (15.1) | 1164 (16.1) | 0.047 | 0.028 |
| Tea intake category, n (%) |  |  |  | 0.948 | 0.008 |
| None | 3705 (15.1) | 2609 (15.1) | 1096 (15.2) |  |  |
| Light | 1822 ( 7.4) | 1284 ( 7.4) | 538 ( 7.4) |  |  |
| Moderate | 6958 (28.4) | 4926 (28.5) | 2032 (28.1) |  |  |
| Heavy | 12016 (49.0) | 8459 (49.0) | 3557 (49.2) |  |  |

*Data are presented as mean ± SD, median [IQR], or n (%). SMD = standardised mean difference; |SMD| > 0.10 indicates meaningful imbalance.*
